# Maternal docosahexaenoic acid and eicosapentaenoic acid supplementation: effects and mechanisms on lipid metabolism in the offspring

**DOI:** 10.3389/fnut.2026.1745358

**Published:** 2026-02-19

**Authors:** Chuhan Shao, Hanmo Lin, Jie Yu, Haiyan Chen, Yaolin Ren, Jing Ren, Yuan Zeng, Yifan Wu, Qian Zhang, Xinhua Xiao

**Affiliations:** Key Laboratory of Endocrinology, Ministry of Health, Department of Endocrinology, Peking Union Medical College Hospital, Peking Union Medical College, Chinese Academy of Medical Sciences, Beijing, China

**Keywords:** DHA, EPA, lactation, lipid metabolism, offspring, pregnancy

## Abstract

Environmental factors, such as nutrition, hormones, and metabolites, which are present in early stages of life, have long-lasting effects throughout an organism’s lifespan, and an abnormal nutritional environment throughout gestation and lactation may significantly increase the possibility that offspring will develop chronic metabolic disorders. The important nutrients docosahexaenoic acid (C22:6n-3, DHA) and eicosapentaenoic acid (C20:5n-3, EPA), which are essential long-chain omega-3 polyunsaturated fatty acids, contribute to proper neurological and retinal development and exhibit both anti-inflammatory properties and lipid-reducing capabilities. Recent research has demonstrated that maternal diets supplemented with EPA and DHA may regulate lipid metabolism-related genes in the liver and adipose tissues and alter the intestinal microbial composition in offspring. These changes influence the progression of lipid metabolic disorders, including dyslipidemia, obesity, and MAFLD in the next generation. This narrative review illustrates the effects of maternal EPA and DHA intervention during the prenatal and breastfeeding period on lipid metabolism in the offspring and the underlying mechanisms. We also explore the directions for future research.

## 1 Introduction

Currently, lipid metabolic disorders, such as dyslipidemia, obesity, and metabolic dysfunction-associated fatty liver disease (MAFLD), pose economic burdens worldwide (1–3). Among them, obesity is traditionally defined as an excess of body fat causing adverse effects on health (1). Dyslipidemia is characterized by increases in plasma low-density lipoprotein (LDL), triglyceride (TG), and total cholesterol (TC) levels or a decrease in high-density lipoprotein (HDL) levels (2). Concurrently, MAFLD has emerged as a revised term for fatty liver disease, which is not a simple renaming of non-alcoholic fatty liver disease (NAFLD), and is diagnosed based on the presence of fatty liver along with overweight/obesity, type 2 diabetes mellitus, or lean/normal weight with evidence of metabolic dysregulation (3).

The latest epidemiological data show that in 2022, 504 million and 374 million women and men were obese, respectively, representing increases of 377 million and 307 million, compared with 1990 (4). Meanwhile, the numbers of obese girls and boys reached 65.1 million and 94.2 million, representing increases of 51.2 million and 76.7 million, respectively, since 1990 (4). According to World Health Organization (WHO) statistics from 2008, elevated total plasma cholesterol affected 39% of the global adult population aged 25 and above (5). Moreover, the current prevalence of MAFLD, according to a large pooled analysis, is estimated to be 39% worldwide (6). Notably, the origins of these diseases may lie in early life, as their development is closely linked to the maternal environment during embryogenesis and lactation, underscoring the necessity of early intervention (7).

The developmental origins of health and disease (DOHaD) theory provides a critical framework for this observation: persistent effect of environmental factors, such as nutrients, hormones, and metabolites, which are present during pregnancy and the initial life course throughout the lifespan of an organism (8). Abnormal nutritional environments throughout gestation and the breastfeeding period significantly increase the incidence of chronic metabolic disorders like obesity, metabolic syndrome, and diabetes mellitus, in offspring during adulthood and even adolescence, via mechanisms such as epigenetic programming (9, 10). Among the conditions, obesity, which is a prominent manifestation of abnormal lipid metabolism, is the most widespread manifestation of transgenerational effects in offspring. Maternal nutrient imbalance may lead to maladaptation of lipid metabolism in the offspring, subsequently triggering chronic conditions like overweight, obesity, and central obesity (8). Therefore, maternal nutrition intervention is recognized as a critical time window for improving the metabolic health of offspring throughout their entire lifespan.

Under such circumstances, long-chain n-3 polyunsaturated fatty acids (PUFAs), particularly docosahexaenoic acid (C22:6n-3, DHA) and eicosapentaenoic acid (C20:5n-3, EPA) have emerged as promising nutrients for modulating lipid metabolism, as well as reducing risk of metabolic diseases (11). N-3 PUFAs show promising effects of lowering TG and non-high-density lipoprotein cholesterol in a recent meta-analysis of RCTs (12). Also, EPA and DHA are regarded as an effective option for managing hypertriglyceridemia according to the American Heart Association (13). N-3 PUFAs regulate lipid metabolism by attenuating lipogenesis, facilitating fatty acid oxidation, promoting intestinal homeostasis, and decreasing liver inflammation and oxidative stress (14). Currently, research has predominantly examined the neurodevelopmental and retino-protective effects of DHA and EPA in offspring. Accumulating evidence in recent years implies that these maternal DHA/EPA supplementation influences lipid metabolic programming in the offspring via transgenerational mechanisms, thereby exerting profound long-term effects on their metabolic health.

Although recent studies have begun to suggest that maternal DHA/EPA supplementation may affect lipid metabolism in adipose tissue, hepatic gene expression, and the intestinal microbiota in the offspring, these findings remain fragmented and unsystematic (15–17). A comprehensive synthesis and interpretation of their transgenerational effects, specific molecular mechanisms, and definitive impacts on offspring’s lifelong metabolic health is still lacking.

Based on this gap, this narrative review aims to systematically address this gap. We aim to integrate existing evidence on the transgenerational effects of maternal DHA/EPA supplementation on offspring short or long-term lipid metabolism, integrating epidemiological, animal, and clinical studies. We will not only summarize their roles in various metabolic organs (such as adipose tissue, liver, gut, and brain) but also emphasize the key underlying mechanisms. The novelty of this review lies in extending beyond single-organ descriptions to reveal network mechanisms of transgenerational lipid metabolic programming and encompassing a broad spectrum of lipid metabolism disorders, thereby providing a theoretical basis for early-life nutritional interventions with n-3 PUFAs to reduce lipid metabolic disorder risks in the offspring.

## 2 Structure, acquisition, functions, and the synthetic pathway of DHA and EPA

N-3 PUFAs are those in which the first methylidene-spaced double bond is located on the third carbon atom at the end of the methyl group of the FA chain, hence named n-3 (18), of which DHA and EPA are two essential representatives that confer cardiovascular and anti-inflammatory benefits while being instrumental in the structural composition of neurons and the retina (19). Although the body produces DHA and EPA on its own, its production of these molecules is very limited. Therefore, dietary supplementation with DHA and EPA is needed, and marine animals are the major source (19).

The functional benefits of DHA and EPA are multifaceted. Notably, EPA and DHA exert inflammation-inhibiting effects in cardiovascular protection (20, 21), in which nuclear factor kappa-B (NF-κB) signaling, hypoxia signaling, scavenger receptor activity, adipogenesis, and eicosanoid production are involved (22). Furthermore, their neuroprotective roles are well-documented. EPA and DHA may demonstrate cognitive benefits in early-stage Alzheimer’s disease (23). In addition, DHA accounts for approximately 25% of total cerebral cortical fatty acids and 50% of the PUFAs in the central nervous system (24). This is supported by 13 RCTs, in which 9 trials reported protective effects of n-3 PUFA supplementation against cognitive decline, whereas 4 trials failed to detect benefits (24). Additionally, maternal DHA supplementation benefits offspring’s early language development (25). Thus, the protective effects of DHA and EPA throughout the lifespan may be observed, including benefits for the normal maturation of the neurological system and the retina during early development, plus the cardiovascular system later in life (26).

**Figure 1:**
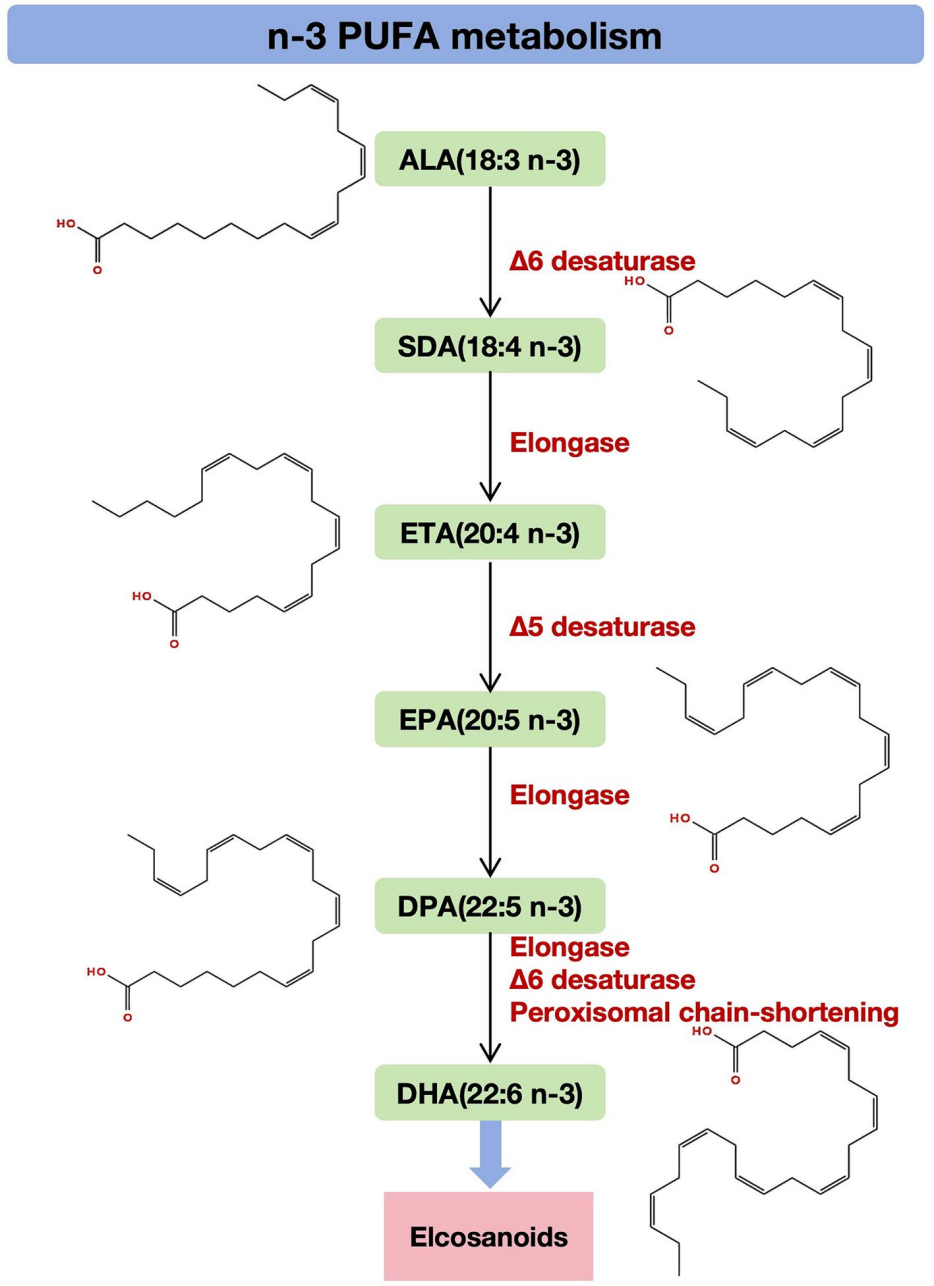
Synthetic pathway of n-3 PUFAs (molecular structure created by https://app.molview.com). The synthetic pathway of ALA to DHA proceeds through a string of enzymatic reactions. Initially, Δ6-desaturase converts ALA to SDA, which is then elongated by elongase to yield ETA. Δ5-desaturase acts on ETA to produce EPA. EPA undergoes further elongation to form n-3 DPA, which is then converted to DHA through the Sprecher pathway. These long-chain n-3 PUFAs ultimately produce eicosanoids. N-3 PUFA, omega-3 polyunsaturated fatty acids; ALA, α-linolenic acid; SDA, stearidonic acid; ETA, eicosatetraenoic acid; EPA, eicosapentaenoic acid; DPA, docosapentaenoic acid; DHA, docosahexaenoic acid.

To understand how these benefits are derived, it is critical to examine the synthetic pathways of DHA and EPA. The precursors of DHA and EPA are a series of n-3 PUFAs. ALA is derived from plants and is processed by elongases and desaturases to produce EPA and DHA and ultimately a range of biologically active compounds called eicosanoids. Specifically, the synthetic pathway of α-linolenic acid (ALA, 18:3n-3) to docosahexaenoic acid (DHA, 22:6n-3) proceeds through a string of enzymatic reactions. Initially, Δ6-desaturase converts ALA to stearidonic acid (SDA, 18:4n-3), which is then elongated by elongase to yield eicosatetraenoic acid (ETA, 20:4n-3). Subsequently, Δ5-desaturase acts on ETA to produce eicosapentaenoic acid (EPA, 20:5n-3). EPA undergoes further elongation to form n-3 docosapentaenoic acid (n-3 DPA, 22:5n-3). DPA is then converted to docosahexaenoic acid (DHA, 22:6n-3) via the Sprecher pathway, involving elongation by elongase, Δ6-desaturation, and peroxisomal chain-shortening through partial β-oxidation (18). These long-chain n-3 PUFAs serve as substrates for enzymes such as cyclooxygenase (COX) and lipoxygenase (LOX), yielding various classes of bioactive eicosanoids (Figure 1) (27, 28).

## 3 Roles of DHA and EPA in lipid metabolism

**Figure 2:**
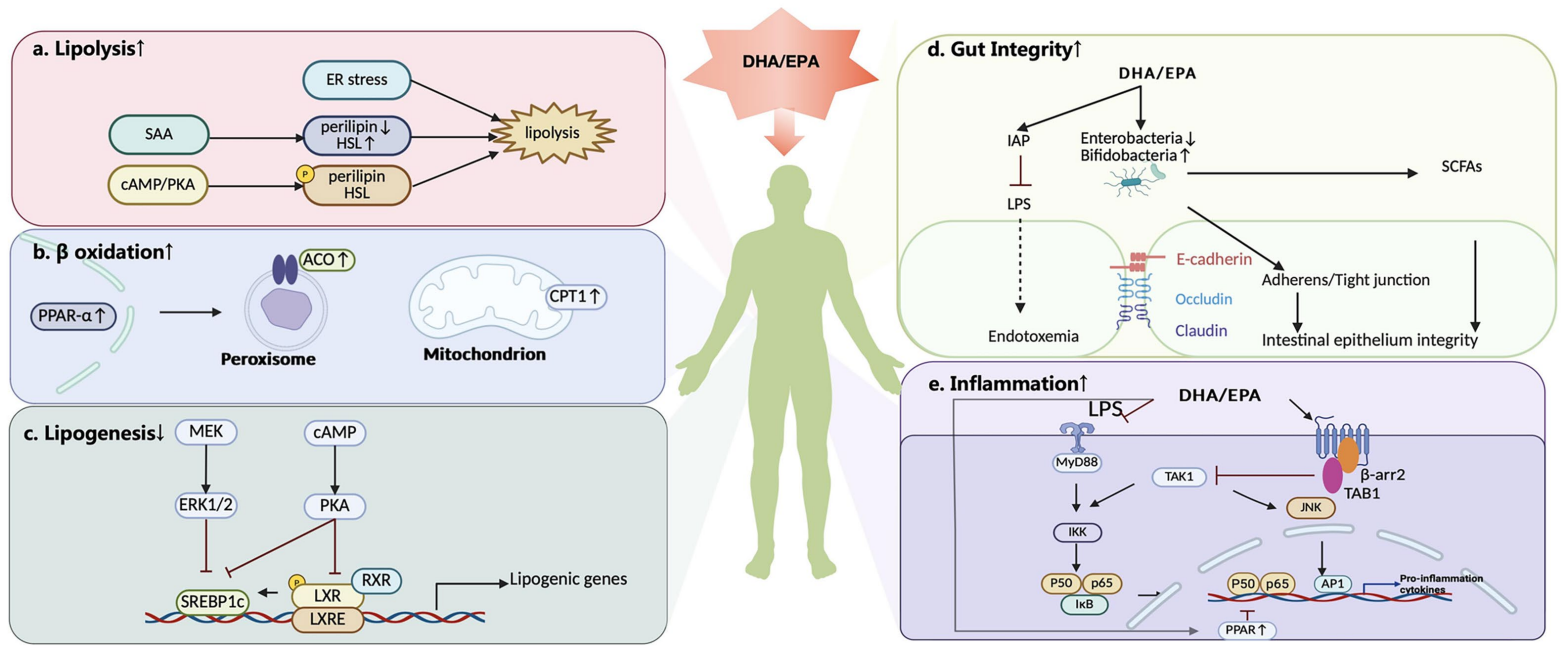
DHA and EPA regulate lipid metabolism through multiple mechanisms, including modulation of lipolysis, β-oxidation, lipogenesis, gut barrier function, and inflammatory pathways (figure created by https://www.biorender.com). **(a)** DHA and EPA stimulate lipolysis. This effect is regulated by ER stress. The cAMP/PKA signaling pathway may partially mediate the lipolytic effects that are induced by DHA and EPA, facilitating phosphorylation mediated by perilipin and HSL. DHA also increases SAA levels, enhancing lipolysis that is mediated by HSL upregulation and perilipin downregulation. **(b)** DHA and EPA may promote β-oxidation by modulating PPAR-α and a variety of metabolic enzymes that play key roles in fatty acid oxidation, including CPT1 and ACO. **(c)** DHA and EPA inhibit lipogenesis, which is mainly orchestrated by SREBP-1. N-3 PUFAs are capable of influencing SREBP-1 expression by tuning the ERK1/2-dependent pathway and PKA activation, and then, the cAMP/PKA pathway further inhibits the interaction of the LXR/RXR heterodimer with LXREs in the SREBP-1c promoter. **(d)** DHA and EPA improve the intestinal environment. DHA and EPA reduce the levels of LPS-producing bacteria, such as *Enterobacteriaceae*, and elevate the proportion of LPS-suppressing microbiota, such as *Bifidobacterium*. DHA and EPA supplementation increase the expression of tight junction proteins in the gut and restore normal cadherin junctions. N-3 PUFA increases the intestinal secretion of IAP, which reduces LPS production by modulating the intestinal microbiota. Furthermore, n-3 PUFA supplementation increases SCFAs-producing bacteria, which protect the intestinal epithelial barrier. **(e)** DHA and EPA regulate inflammation to affect lipid metabolism. Studies have demonstrated that DHA and EPA suppress proinflammatory factors. DHA suppresses the NF-κB pathway that is activated by the TLRs. EPA and DHA inhibit TAK1 in a β-arrestin 2/TAB1-dependent manner, which reduces the activity of the IKK-β/NF-κB and JNK/ AP-1 signaling pathways. EPA and DHA also inhibit inflammation by activating PPARs, which inhibit NF-κB. DHA, docosahexaenoic acid; EPA, eicosapentaenoic acid; ER stress, endoplasmic reticulum stress; SAA, serum amyloid A protein; cAMP, cyclic adenosine monophosphate; PKA, protein kinase A; HSL, hormone-sensitive lipase; PPAR-α, peroxisome proliferator-activated receptor alpha; CPT1, carnitine palmitoyl transferase-1; ACO, acyl-CoA oxidase; SREBP-1, sterol regulatory element binding protein-1; ERK1/2, extracellular signal-regulated kinase 1/2; LXR, liver X receptor; RXR, retinoid X receptor LXREs, LXR response elements; IAP, intestinal alkaline phosphatase; LPS, lipopolysaccharide; SCFAs, short-chain fatty acids; β-arr2, β-arrestin 2; TAK1, transforming growth factor β-activated kinase 1; TAB1, transforming growth factor β-activated kinase 1 binding protein; IKK-β, inhibitor of nuclear factor kappa-B kinase subunit β; JNK, c-Jun N-terminal kinase; AP1, activator protein-1.

DHA and EPA may regulate lipid metabolism by stimulating lipolysis and β-oxidation, attenuating lipogenesis and inflammation, and exerting a beneficial effect on the gut barrier (27, 29–34) (Figure 2).

Firstly, these fatty acids may stimulate lipolytic activity (29, 30). This effect may involve the regulation of endoplasmic reticulum stress (29). Additionally, the cyclic adenosine monophosphate (cAMP)/protein kinase A (PKA) signaling pathway appears to partially mediate the lipolytic effects that are induced by n-3 PUFAs, facilitating phosphorylation mediated by perilipin and hormone-sensitive lipase (HSL) (29, 35–37). DHA also increases serum amyloid A protein (SAA1) levels, enhancing lipolysis that is mediated by HSL upregulation and perilipin downregulation (30).

In contrast to promoting fat breakdown, DHA and EPA may concurrently inhibit lipogenesis, which is mainly orchestrated by sterol regulatory element binding protein-1 (SREBP-1). N-3 PUFAs inhibit SREBP-1, thereby suppressing stearoyl-CoA desaturase-1 (SCD), fatty acid synthase (FAS), and the lipogenic genes acetyl-CoA carboxylase (ACC) (38, 39). Plus, n-3 PUFAs are capable of influencing SREBP-1 expression by tuning the extracellular signal-regulated kinase 1/2 (ERK1/2)-dependent pathway (31) and PKA activation (40). Subsequently, the cAMP/PKA pathway further inhibits the interaction of the liver X receptor (LXR)/retinoid X receptor (RXR) heterodimer with LXR response elements (LXREs) in the SREBP-1c promoter (40, 41).

N-3 PUFAs may also promote β-oxidation by modulating peroxisome proliferator-activated receptor alpha (PPAR-α) and a variety of metabolic enzymes that play key roles in fatty acid oxidation, including carnitine palmitoyl transferase-1 (CPT1), acyl-CoA oxidase (ACO) (27). PPAR-α transcriptionally governs the expression of fatty acid oxidation enzymes in the liver. Notably, n-3 PUFAs activate PPAR-α in both hepatic and adipose tissues (42, 43), thereby activating the mitochondrial and peroxisomal β-oxidation pathways (44).

Beyond direct metabolic regulation, DHA and EPA may also improve the intestinal environment and protect against metabolic disorders induced by a high-fat diet (HFD) (32). A HFD introduces gut microbiota aberrations and damages intestinal barrier integrity, promoting the translocation of harmful substances into systemic circulation and triggering metabolic dysregulation (45). On the one hand, a HFD leads to gut dysbiosis. Abnormal *Firmicutes/Bacteroidetes* ratios are observed to be correlated with obesity, insulin resistance, and increased intestinal permeability (46). A HFD elevates the levels of LPS-producing bacteria, such as *Enterobacteriaceae*, and reduces the proportion of LPS-suppressing microbiota, such as *Bifidobacterium* (28, 47). DHA and EPA alleviate this change and the inflammatory response associated with metabolic endotoxemia (48). Conversely, HFD feeding leads to a compromised intestinal barrier, whereas DHA and EPA supplementation increases the expression of tight junction proteins in the gut (49) and restores normal cadherin junctions (48). N-3 PUFAs increase the intestinal secretion of intestinal alkaline phosphatase (IAP), which reduces LPS production by modulating the intestinal microbiota (50). Furthermore, n-3 PUFA supplementation increases the amount of bacteria that may reversibly produce short-chain fatty acids (SCFAs) (51), which protect the intestinal epithelial barrier (48).

Finally, the anti-inflammatory properties of DHA and EPA are integral to their lipid-modulating effects. HFD-induced inflammation is a key driver of metabolic dysfunction. Studies have demonstrated that DHA and EPA suppress proinflammatory factors, including COX-2, iNOS, and IL-1 (33, 34). Toll-like receptors (TLRs) serve as key mediators of inflammatory reactions in individuals with obesity (52). DHA suppresses the NF-κB pathway that is activated by the TLRs (53), thus decreasing cytokine and COX-2 expression (34). Additionally, EPA and DHA modulate G protein-coupled receptor 120 (GPR120)-mediated anti-inflammatory effects by inhibiting transforming growth factor β-activated kinase 1 (TAK1) in a β-arrestin 2/transforming growth factor β-activated kinase 1 binding protein (TAB1)-dependent manner, which reduces the activity of the inhibitor of nuclear factor kappa-B kinase subunit β (IKK-β)/NF-κB and c-Jun N-terminal kinase (JNK)/activator protein-1 (AP-1) signaling pathways (54, 55). In addition, EPA and DHA reduce inflammation by activating GPR120/PPARγ signaling pathway (56, 57). EPA and DHA also inhibit inflammation by activating PPARs, which inhibit NF-κB (58). The reduction in oxidative stress caused by n-3 PUFAs may be achieved through immunomodulation and a reduction in leukocyte activation (59).

## 4 Roles of DHA and EPA in lipid metabolic disorders

### 4.1 Effects of DHA and EPA on obesity

In animal models, EPA and DHA have been shown to exert beneficial effects against obesity. For instance, animals that are fed a DHA or EPA diet have been shown to experience weight loss or decreased adipose tissue mass (60–62). Specifically, HFD-fed mice that received 1% DHA or 4% EPA supplementation exhibited body weight loss (60). Furthermore, in another research, the administration of EPA/DHA reduces visceral adipocyte size as well as adiposity induced by HFD (61). Supplementation with EPA and DHA in the context of a HFD reduced lipid accumulation in both brown adipose tissue (BAT) and white adipose tissue (WAT) in C57BL/6J mice (63). These positive effects are attributed to the fact that EPA and DHA benefit lipid homeostasis, adipocyte function, and leptin and adiponectin production (62).

Although DHA and EPA supplementation pose beneficial effects for obesity in animal models, this effect remains rather multidimensional in humans (64, 65). Meta-analyses have shown that n-3 PUFA does not affect weight loss or BMI; however, it may provide benefits by reducing waist circumference and TG level in obese adults (64, 65).

### 4.2 Effects of DHA and EPA on MAFLD

According to the Global Burden of Disease 2019 report, a diet low in n-3 PUFAs is associated with an elevated risk of mortality from MAFLD (66). Clinically, recent meta-analyses showed that the marine-based n-3 PUFAs significantly reduce alanine aminotransferase (ALT), aspartate aminotransferase (AST), and γ-glutamyl transferase (GGT) levels (67). Moreover, another meta-analysis demonstrated significant improvement in liver fat content after taking marine-based n-3 PUFAs (68). This benefit is also observed in younger populations. Another meta-analysis focusing on pediatric MAFLD showed that n-3 PUFA supplementation combined with health behavior adjustment had a positive effect on ALT, AST, and GGT levels (69). It was also suggested that dietary C20–22 n-3 PUFA supplementation may decrease MAFLD severity by reducing steatosis (70).

### 4.3 Effects of DHA and EPA on dyslipidemia

Currently, EPA alone or combined with DHA is considered a well-tolerated and effective agent for treating hypertriglyceridemia, and according to a recent advisory from the American Heart Association, prescription n-3 LCPUFAs at a dose of 4 g/day is recommended as a triglyceride-lowering option (13). The TG-lowering mechanism of DHA and EPA is multifactorial, involving the suppression of hepatic TG synthesis, inhibition of TG incorporation into VLDL particles, reduction of TG secretion, and promotion of TG clearance from VLDL, collectively leading to reduced plasma TG concentrations (71). Evidence from meta-analyses robustly supports this effect. Meta-analysis showed that n-3 PUFA supplementation lowered blood TG, but effects on HDL remain inconsistent (72). Similar promising effects of lowering TG and non-high-density lipoprotein cholesterol are shown in another recent meta-analysis of RCTs (12).

## 5 Roles of maternal DHA and EPA supplementation in transgenerational lipid metabolism

**Figure 3:**
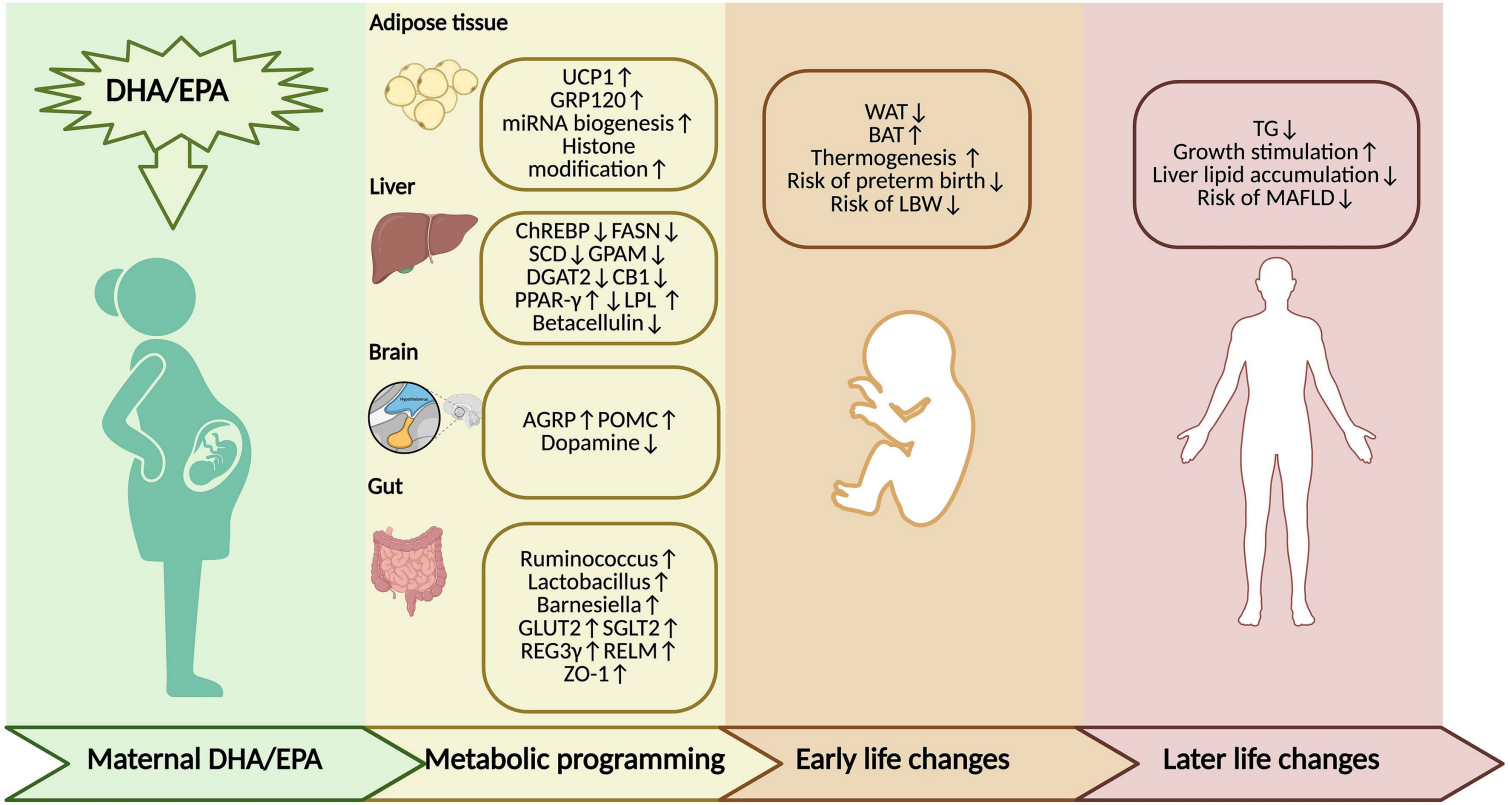
The roles and critical windows of maternal DHA and EPA supplementation in transgenerational lipid metabolism regulation (figure created by https://www.biorender.com). Maternal supplementation of DHA and EPA regulates metabolic programming. Upregulation of UCP1, GPR120, miRNA biogenesis, and histone modification is observed in brown adipose tissue. Downregulation of ChREBP, FASN, SCD, GPAM, DGAT2, CB1, and Betacellulin, as well as upregulation of LPL, is observed in offspring liver; however, PPAR-γ regulation shows inconsistent results. AGRP and POMC are upregulated in the hypothalamus, while dopamine is reduced in the offspring’s brain. Maternal DHA/EPA supplementation increases the abundance of beneficial gut microbiota, including *Ruminococcus, Lactobacillus*, and *Barnesiella*, and increases mucosal integrity markers (Relmβ, REG3γ, and ZO-1) in the gut. In addition, genes involved in carbohydrate transport, such as GLUT2 and SGLT1, are upregulated in the offspring. These mechanisms induce early-life changes characterized by reduced WAT but increased BAT accumulation, enhanced thermogenic capacity, and a lower risk of preterm birth and low birth weight. In later life, they are associated with stimulated growth and decreased triglyceride levels, reduced hepatic lipid accumulation, as well as lower risk of MAFLD. EPA, eicosapentaenoic acid; DHA, docosahexaenoic acid; PUFA: polyunsaturated fatty acid; WAT, white adipose tissue; BAT, brown adipose tissue; UCP1, uncoupling protein 1; GPR120, G protein-coupled receptor 120; miRNA, microRNA; ChREBP, carbohydrate response element binding protein; FASN, fatty acid synthase; SCD, stearoyl-CoA desaturase; GPAM, glycerol-3-phosphate acyltransferase, mitochondrial; DGAT2, diacylglycerol O-acyltransferase 2; CB1, cannabinoid receptor type 1; LPL, lipoprotein lipase; PPAR-γ, peroxisome proliferator-activated receptor gamma; AGRP, agouti-related peptide; POMC, pro-opiomelanocortin; GLUT2, glucose transporter type 2; SGLT1, sodium-glucose cotransporter 1; MAFLD, metabolic dysfunction-associated fatty liver disease.

Maternal DHA and EPA supplementation has transgenerational effects on offspring throughout their lifespan (Figure 3). A total of 16 clinical studies, alone with 4 meta-analyses and 14 animal studies, have examined the link between maternal DHA and EPA exposure and the subsequent lipid metabolism in the offspring (Tables 1, 2).

**Table 1:** Clinical studies and meta-analysis of maternal DHA/EPA supplementation affecting the offspring.

Country	Participant	Intervention	Maternal DHA/EPA status	Age of offspring	Influence on mothers and offspring
Mexico (98)	1,094 pregnant women	DHA 400 mg/day	NA	4 years offspring	Non-fasting serum lipid and glucose concentrations of offspring not affected
America (77)	350 pregnant women	DHA 600 mg/day	Baseline: Mean RBC DHA is low in both groups Maternal RBC DHA: DHA: 4.3 (1.1) vs. Placebo: 4.3 (1.3); (% total fatty acids) During gestation: NA Post-intervention: Maternal RBC DHA: DHA: 7.3 (2.2) vs. placebo 4.7 (1.3) (% total fatty acids)	Newborn	Gestation duration↑ Birth weight↑ Early preterm and very-low birth weight↓
Denmark (99)	700 pregnant women	2.4 g n-3 LCPUFA (55% EPA & 37% DHA)	Baseline: No significant interaction between baseline blood concentrations and the intervention on either anthropometrics or metabolic measurements During gestation: NA Post-intervention: NA	10 years offspring	BMI↑ OR of being overweight ↑
Denmark (82)	736 pregnant women	2.4 g n-3 LCPUFA (55% EPA & 37% DHA)	Baseline: Whole blood concentrations of EPA + DHA: n-3 LCPUFA: 4.9 (1.3); Placebo: 4.9 (1.2) (% measured blood fatty acids) No significant interaction between baseline blood concentrations and the intervention on either anthropometric outcomes During intervention: NA Post-intervention: NA	From 1 week to 6 years of age	**1 year to 6 years of age** BMI↑ BMI *z* score↑ **6 years offspring** Waist circumference↑ Total mass↑ Lean mass↑ Fat mass↑ Risk of obesity not affected
Denmark (102)	533 pregnant women	2.7 g n-3 LCPUFA	NA	19 years offspring	BMI, waist circumference not affected
Denmark (100)	533 pregnant women	2.7 g n-3 LCPUFA	NA	19 years offspring	Plasma lipids, lipoproteins not affected
New Zealand (80)	Overweight or obesity pregnant women	6 g fish oil (3.55 g of n-3 PUFAs)	Baseline: Maternal RBC DHA + EPA: Fish oil: 7.11 (1.55) vs. Placebo: 7.21 (1.49) (% total fatty acids) During intervention (30 weeks of gestation): Maternal RBC DHA + EPA: Fish oil: 10.43 (2.25) vs. Placebo: 6.90 (1.55) (% total fatty acids) Post-intervention: NA	2 weeks offspring 3 months offspring	**2 weeks offspring** Infant body fat percentage not affected **3 months offspring** BMI *z*-score↑ Ponderal index↑ TG↓ Body fat percentage not affected
INFAT (101)	208 pregnant and lactating women	1,200 mg LCPUFAs (1,020 mg DHA + 180 mg EPA + vitamin E 9 mg)	NA	5 years offspring	Skinfold thickness, subcutaneous and visceral adipose tissue volumes and ratio not affected
America (81)	72 Obese/GDM women	DHA 800 mg/day	Baseline: Maternal RBC DHA: DHA: 5.8 (2.1) vs. Placebo: 6.0 (2.2) (% total fatty acids) During intervention (36 weeks of gestation): Maternal RBC DHA: DHA: 9.7 (2.6) vs. Placebo: 6.2 (2.2) (% total fatty acids) Post-intervention: NA	Newborn 2 years offspring 4 years offspring	**Newborn** Birth weight, length not affected **2 years 4 years** BMI, weight, height, arm circumference, arm skinfold *z*-scores not affected
Australia (103)	1,660 children of singleton pregnant women	DHA 800 mg/day + EPA 100 mg/day	NA	3 years offspring 5 years offspring	**3 years and 5 years** BMI *z* score, percentage of body fat, Hip and waist circumferences, waist-circumference *z* scores, body weight and height *z* scores, fat-free mass, the percentage fat-free mass not affected **3 years** Waist: hip ratio↑ **5 years** HOMA-IR↑ Fasting insulin concentrations↑
America (78)	350 pregnant women	DHA 600 mg/day	Baseline: Maternal RBC DHA: DHA: 4.4 (1.1) vs. Placebo: 4.4 (1.2) (% of total fatty acids); During intervention: NA Post-intervention: Maternal RBC DHA: DHA: 7.7 (2.0) vs. Placebo: 4.7 (1.1) (% of total red blood cell fatty acids)	5 years offspring	Fat mass, fat-free mass, body fat, height, weight, BMI *z* score not affected
America (89)	1,100 pregnant women on early preterm birth <34 weeks gestation	DHA 200 or 1,000 mg/day	NA	Newborn	Risk of preterm birth ↓ Birth weight ↑
Mexico (104)	1,040 pregnant women	DHA 400 mg/day	NA	60 months offspring	Weight, height, BMI, height-*z* score, weight-*z* score, BMI-for-age *z* score not affected
Germany (105)	144 pregnant women	DHA 200 mg + EPA 60 mg	NA	6 years offspring	Weight, BMI, and skin-fold thickness not affected BMI z-score ↑
Germany (79)	208 pregnant women	1,200 mg LCPUFAs (DHA 1,020 mg + EPA 180 mg + vitamin E 9 mg)	Baseline: Maternal RBC DHA: fish oil: 4.55 (1.61) vs. Placebo: 4.54 (1.24) (% of total fatty acids) Maternal RBC EPA: fish oil: 0.42 (0.18) vs. Placebo: 0.42 (0.15) (% of total fatty acids) During intervention (32 weeks of gestation): Maternal RBC DHA: fish oil: 7.18 (2.97) vs. Placebo: 4.34 (2.07) (% of total fatty acids) Maternal RBC EPA: fish oil: 0.66 (0.32) vs. Placebo: 0.33 (0.16) (% of total fatty acids) Post-intervention: NA	1 year offspring	Skinfold thickness, abdominal fat mass, fat distribution not affected
India (96)	957 pregnant women	DHA 400 mg/day	Baseline: Maternal RBC DHA: DHA: 0.86 (0.78) vs. Placebo: 0.88 (0.71) (mol% of total red blood cell fatty acids) During intervention: NA Post-intervention: Maternal RBC DHA: DHA: 2.03 (1.76) vs. Placebo: 1.12 (0.86) (mol% of total red blood cell fatty acids)	Newborn	Birth weight, length not affected
Meta-analysis (83)	Pregnant women	DHA and/or EPA supplementation (various levels)	NA	Newborn, 0–4 years, 5–10 years	Birth weight↑ (doses >650 mg/day) BMI *z* score↑ (5–10 years)
Meta-analysis (84)	Pregnant women	Algal DHA, DHA, EPA + DHA	NA	Offspring	BMI, BMI *z* score, skinfold thickness, body fat (%), fat mass, weight, height, weight *z* score, height *z* score not affected
Meta-analysis (85)	19,927 women at low, mixed or high risk of poor pregnancy outcomes	n-3 LCPUFA (supplements and food)	NA	Newborn	Risk of LBW babies ↓LGA babies ↑ Early preterm birth <34 weeks ↓ Preterm birth <37 weeks↓
Meta-analysis (86)	Pregnancy and/or lactation women	DHA/EPA supplementation	NA	Newborn or child	**Newborn** Birth weight↑ Waist circumference↑ Birth length not affected **Child** Postnatal length, postnatal weight, BMI, the sum of skinfold thicknesses, fat mass, body fat (%) not affected

LBW, risk of low birthweight; LGA, large-for-gestational-age; OR, odd ratio; BMI, body mass index; HOMA-IR, homeostatic model assessment of insulin resistance; TG, triglyceride. The bold formatting is used to distinguish different subgroups, particularly by age, mechanisms, or affected tissues.

**Table 2:** Animal studies of maternal DHA/EPA supplementation affecting the offspring.

Animal model	Intervention	Dose	Intervention phase	Age of offspring	Influence on offspring	Mechanism
HFrD ddY mice (97)	FO (DHA-22K)	4% DHA-22K (EPA 5.1% + DHA 27.3% of fatty acid composition of DHA-22 K)	During pregnancy	5 days	Body weight↑ **Liver** Liver weight (% body weight) ↑ Hepatic lipid accumulation not affected **Plasma** TG↓ FFA↑ T-Cho↑	**T-Cho synthesis** Hmgcr and Hmgcs1↑ in the liver
Maternal HF diet Wistar rats (91)	FO	3%FO (DHA 1,500 mg/100 g + EPA 600 mg/100 g of HFFO diet)	During pregnancy	Newborn	Birth weight↓ **Liver** Liver TG↓(F) liver mass not affected	**ECS activation↓** CB1↓AEA↓2-AG↓ CB2↓(F) in the liver **Lipogenic marker** Srebf1c↓ in the liver
Maternal HF diet Wistar rats (95)	FO	2.9% w/w FO (DHA 1.46% + EPA 0.58% of fatty acid composition of HFFO diet)	During pregnancy	21 days	Birth weight not affected Inguinal mass↑(F) **Plasma** Plasma TG not affected **Liver** Liver steatosis (M↑F not affected) Ballooning↑ (M, F) Inflammation↑(M↑ F↓)	**Lipogenic markers** Srebf1c↓ Fasn↓ in the liver
C57BL/6 mice (15)	FO	3% FO (DHA 12.70% + EPA 13.36% of fatty acid composition of fish oil diet)	During pregnancy and lactation	3 weeks	Energy expenditure↑ Maintenance of core body temperature↑ Fetal BAT development potential↑	**Histone modifications** H3K27Ac↑ H3K9me2↑ in BAT **miRNA production** miR-30b↑ miR-193b/365↑ Drosha↑ in BAT
C57BL/6J mice (87)	FO enriched with EPA and DHA (FA)	3.05% FO (EPA 5.7% + DHA 4.5% of fatty acid composition of FA diet)	During pregnancy and lactation	Fetus of 13 days, offspring 1 day, 21 days	Body weight not affected **Adipose tissue** BAT development and activity↑ Browning of WAT↑	**D21** **Lipolysis** ATGL↑ HSL↑MGL↑ LPL↑ in liver **β-oxidation and thermogenesis** CPT1α↑ Ehhadh↑ Mcad↑ Lcad↑ Acadvl↑ Slc22a5↑ Slc25a20↑ PPARα↑ in liver **BAT activity** Ucp1↑ Cidea↑ Prdm16↑ PGC1α↑ Dio2↑ Zic1↑ Fgf21↑ p2rx5↑ PPARα↑ in BAT **Beige specific markers** Ucp1↑ Shox2↑ Tmem26↑ Pat2↑ in subcutaneous fat
Wistar rats (16)	FO	EPA 77 mg/g + DHA 521 mg/g of FO	During pregnancy and lactation	3 months, 6 months	**Obese offspring** **3 weeks** Body weight, fat accretion and hyperlipidemia not affected **6 months liver** TG↓(M, F) Cholesterol↓(M)	**β-oxidation** PPAR-γ↓CPT1a↓PGC-1α↓MCAD↓HADH ↓ in liver **Lipogenic markers** ChREBP↓FASN↓SCD↓GPAM↓DGAT2↓ in liver
C57BL/6J mice (108)	Menhaden oil	30 g/kg FO (EPA 14% + DHA 11% of fatty acid composition of fish oil diet)	During pregnancy and lactation	21 days before weaning+ (PW 13 weeks)	**FO–FO & FO-HF vs. HF-FO & HF–HF** **Liver** Tg levels ↓(M) **FO–FO vs. HF–HF** Adipocyte size↓ Glucose clearance↑ Insulin sensitivity↑	**Inflammation** Mcp1↓in gonadal fat and liver
Maternal high-fructose diet rats (115)	FO	2.5% FO (9.05% EPA 11.59% DHA of fatty acid composition of high-fructose-DHA diet)	During pregnancy	24 h	**Liver** Oleic acid, saturated fatty acids, linoleic acid and n3-docosapentaenoic acid↓	Δ-9 desaturation↓ Hepatic betacellulin↓ in liver
C57BL/6 mice (124)	FO	3% menhaden fish oil (EPA 4.22% + DHA 3.10% of fatty acid composition of menhaden fish oil diet)	During pregnancy	21 days	V/C ratio↑ Goblet cell↑ Crypt lengths↑	**Mucosal integrity** Relmβ, REG3γ, ZO-1↑ in gut
Wistar rats (119)	ω3 diet	EPA 0.27% + DHA 1.19% of fatty acid composition of ω3 diet	During pregnancy	24 h	Weight↓ Circulating leptin↓	**Hypothalamic neuropeptides** Agrp and Pomc↑(Female) in brain
C57BL/6 mice (17)	DHA (purity >98%)	L-DHA 150 mg/kg/day H-DHA 450 mg/kg/day (750 and 2,250 mg per day for humans)	During pregnancy and lactation	21 days	**Exercise performance** Grip strength↑ swimming duration↑ **Intestine** Villus height↑ Surface area↑ Beneficial gut bacteria abundance↑ V/C ratio↑ Intestinal glucose absorption↑	**Glucose absorption** GLUT2↑SGLT1↑mTOR pathway↑ in gut
Sprague–Dawley rats (92)	FO	10% FO (EPA 6.95 mg/100 mg + DHA 7.72 mg/100 mg of fatty acid composition of fish oil diet)	During pregnancy and lactation	Fetuses of 20 days Newborn: 1 day, 10 days, 20 days, 30 days	Birth weight↓ **1 day** **Plasma** Plasma TG↓ Unesterified fatty acids↓ 3-hydroxybutyrate↓ **Liver** Liver TG↓	**PUFAs synthesis** Δ6-desaturase↓ in liver **Lipogenesis** SREBP-1c↓ in liver **Oxidation** CPT I↑ ACO↑ HMG-CoA synthase↑ in liver **Lipolysis** LPL↑ in liver
Wistar rats (94)	n-3 LCPUFA (high omega-3)	1.29% n-3 LCPUFA ∼15 mg/kg/day (EPA 0.34% + DHA 0.95% of fatty acid composition of high omega-3 diet)	During pregnancy and lactation	0–6 weeks	Birth weight not affected **6 weeks** Total percentage body fat↑ Subcutaneous fat mass ↑	**N/A**
C57BL/6J mice (139)	Menhaden oil enriched with EPA	Menhaden oil enriched with EPA 26 g/kg	During pregnancy and lactation	4 weeks before weaning + (PW 12 weeks)	**FO-HF vs. HF–HF** Glucose tolerance not affected (M)	**FO-HF vs. HF–HF** **Oxidation** Foxa2↑ Cpt2 ↑ in adipose tissue Foxa2↑ Pparα↑ in liver

M, male; F, female; h, hour; d, day; w, week; m, month; HFrD, high-fructose diet; FO, fish oil; pw, post weaning; FFA, free fatty acid; T-Cho, total cholesterol; TG, triglyceride; Hmgcr, 3-hydroxy-3-methylglutaryl-coenzyme A reductase; Hmgcs1, 3-hydroxy-3-methylglutaryl-coenzyme A synthase 1; HF, high-fat diet; ECS, endocannabinoid system; SREBP-1c/Srebf1c, sterol regulatory element-binding protein-1c; CB1, cannabinoid receptor 1; CB2, cannabinoid receptor 2; AEA, anandamide; 2-AG, 2-arachidonoylglycerol; w/w, weight by weight; Fasn, fatty acid synthase; BAT, brown adipose tissue; WAT, white adipose tissue; ATGL, adipose triglyceride lipase; HSL, hormonesensitive lipase; MGL, monoacylglycerol lipase; LPL, lipoprotein lipase; Mcad, medium-chain acyl-CoA dehydrogenase; Lcad, acyl-CoA dehydrogenase, long chain; Acadvl, very long-chain specific acyl-CoA dehydrogenase; Slc25a20, mitochondrial carnitine/acylcarnitine carrier protein; SLC22A5, solute carrier family 22 member 5; Cpt1α, carnitine palmitoyl transferase 1A; Ehhadh, enoyl-CoA hydratase and 3-hydroxyacyl CoA dehydrogenase; Ucp1, uncoupling protein 1; Cidea, cell death-inducing DNA fragmentation factor α-like effector A; Prdm16, PR domain containing 16; PGC1α, peroxisome proliferator-activated receptor gamma coactivator 1-alpha; Dio2, type II iodothyronine deiodinase; Zic, zinc finger protein; Fgf21, fibroblast growth factor 21; p2rx5, P2X purinoceptor 5; PPARα, peroxisome proliferator-activated receptor alpha; Shox2, short-stature homeobox 2; Tmem26, transmembrane protein 26; Pat2, phosphateacetyltransferase; ChREBP, carbohydrate response element binding protein; SCD, stearoyl-coenzyme A desaturase; GPAM, glycerol-3-phosphate acyltransferase, mitochondrial; DGAT2, diacylglycerol O-acyltransferase 2; PPAR, peroxisome proliferator-activated receptor; PGC-1α, peroxisome proliferator-activated receptor γ coactivator 1α; HADH, hydroxyacyl-coenzyme A dehydrogenase; MCP1, monocyte chemoattractant protein 1; PFO, EPA and DHA-rich fish oil; DHAGF, DHA gold fat; SGLT1, sodium glucose co-transporter 1; GLUT2, glucose transporter 2; AMPK, AMP-activated protein kinase; mTOR, mammalian target of rapamycin; V/C ratio, villous height to crypt depth ratio; REG3γ, regenerating family member gamma; Relmβ, resistin-like molecule β; ZO-1, zonula occludens; AgRP, agouti-related peptide; POMC, pro-opiomelanocortin; CPT1, carnitine palmitoyl transferase-1; ACO, acyl-CoA oxidase; HMG-CoA synthase, 3-hydroxymethyl glutaryl-CoA synthase; Foxa2, forkhead box A2; Cpt2, carnitine palmitoyltransferase 2; NEFA, non-esterified fatty acid.

Specifying whether maternal DHA/EPA status has been assessed in a clinical setting is crucial for determining whether supplementation is needed to meet nutritional recommendations (73), especially at enrollment (baseline), during the intervention, or at the end of supplementation. The most common method to assess maternal DHA/EPA levels is to measure their percentage in total red blood cell (RBC) fatty acids (74). Although a consensus on the optimal blood DHA or EPA level during pregnancy has not yet been reached, it has been suggested that if a pregnant woman is deficient in DHA/EPA (<5% RBC DHA, equivalent to 5.9% RBC EPA + DHA) (74), a daily intake of at least 600 to 800 mg of DHA is recommended. If her RBC DHA level is above 5%, she should be encouraged to maintain her current dietary and supplementation habits for the remainder of the pregnancy, including meeting the current recommended daily intake of at least 200 mg of DHA. Maintaining higher RBC DHA levels (RBC DHA 6.5–8%, equivalent to RBC EPA + DHA of 8–12%) appears to be safe and likely more desirable (75). Furthermore, for total omega-3 fatty acids in whole blood (expressed as a percentage of total fatty acids), a level below 4.2% is generally considered low (76).

Among 16 clinical studies included in this review, 8 of which measured maternal DHA/EPA levels either at baseline, during the intervention, or post-intervention. Among these eight studies, seven provided specific data. At baseline, most studies reported low maternal DHA and EPA levels (77–79). Following intervention, the supplementation group showed a significantly higher DHA/EPA status, predominantly above 5%, compared to the placebo group, which mostly remained below 5% (77–79). This suggests that DHA/EPA intervention is an effective strategy for raising RBC DHA/EPA levels and is necessary to correct deficiencies and achieve sufficient nutritional status. The baseline level of maternal omega-3 is sufficient in two studies (80, 81). Even when mothers start with deficient baselines and achieve sufficient status after supplementation, offspring outcomes across studies are inconsistent: some research shows stimulated growth with increased weight (including both lean and fat mass) (82), while others report no effect on weight or body fat (78, 79). A similar pattern of mixed results is observed when mothers begin with already adequate baselines (80, 81). Given the limited number of studies that have systematically investigated baseline omega-3 status, it is premature to draw definitive conclusions based solely on whether participants were initially sufficient or deficient. Also, the four included meta-analyses did not specify whether maternal omega-3 fatty acid status was assessed during pregnancy. Consequently, future meta-analyses that pay closer attention to detailed subgroup analyses based on maternal omega-3 status at baseline and/or achieved levels will be of great scientific and clinical interest (83–86).

Additionally, current studies have primarily focused on short-term effects during infancy and childhood, and have not yet provided sufficient data on the long-term effects of maternal DHA/EPA exposure in either adolescence or adulthood. Furthermore, the existing clinical and preclinical research has predominantly focused on the effects of DHA supplementation, or the combined administration of DHA and EPA, leaving the specific and isolated effects of EPA supplementation relatively underexplored. The distinct transgenerational impacts of DHA compared with EPA are largely unexplored. Here, we illustrate the transgenerational effects of maternal DHA and EPA supplementation on offspring, spanning from the fetal stage to long-term distant impacts.

### 5.1 Influence on lipid metabolism during the fetal period

Maternal DHA and EPA supplementation exerts transgenerational effects on offspring during the fetal period. These transgenerational effects of maternal n-3 PUFA supplementation on fetal adipogenesis and thermogenesis have been investigated in animals only. Prenatal n-3 PUFA supplementation results in a decrease in fetal white fat accumulation, an accumulation of brown fat, and an increase in thermogenesis (15, 87). In contrast, a deficiency in maternal n-3 PUFAs affects the levels of growth hormone and fetal thermogenic-sensitive adipose tissue, as well as energy balance, in offspring, resulting in the body fat accumulation in offspring (88). Moreover, such a deficiency dysregulates differentiation during BAT development and downregulates uncoupling protein 1 (UCP1); since reduced expression of UCP1 reflects insufficient brown fat generation caused by an impairment of thermogenic fat development, this might lead to a higher risk of obesity later in life (88).

Furthermore, maternal n-3 PUFA supplementation promotes the development of brown fat production in the fetus and confers lasting thermogenic benefits to the offspring. Maternal n-3 PUFA supplementation reduced fetal white fat accumulation and promoted brown fat development. As for thermogenic potential, after the cessation of breastfeeding, both groups consumed an n-3 PUFA-free diet. At 11 weeks of age, exposure to cold increased the expression of UCP1 and PPARγ in the BAT and WAT of the fish oil group, increasing the rate of energy dissipation. Thus, maternal n-3 PUFA supplementation not only maintained stronger thermogenic potential in BAT but also was associated with increased adaptive thermogenesis in WAT, conferring sustained thermogenic advantages for offspring (15).

At the level of WAT-to-BAT conversion, maternal DHA and EPA upregulated genes that are associated with brown adipogenesis, as did that of thermogenic stimulators in the offspring. Notably, these changes occurred without changing the adipose tissue mass, suggesting a potential browning effect on WAT (87).

### 5.2 Short-term metabolic effects

The results of animal and clinical studies related to how maternal DHA and EPA supplementation affects neonatal birth weight are inconsistent. On the one hand, prenatal DHA supplementation prolongs gestation and increases newborn body weight (77). DHA and EPA supplementation during pregnancy prolongs gestation and increases birth weight in offspring, and this increase may be safe and beneficial in reducing the risk of preterm birth and the risk of low birth weight (LBW) in infants (77, 83, 84, 86). Another study also revealed a reduced risk of preterm birth and an increase in birth weight (89). The rise in the birth weight of offspring following DHA and EPA supplementation during pregnancy may be related to a prolongation of gestation by affecting endogenous prostaglandin metabolism (86). N-3 LCPUFAs reduce the activity of the labor-promoting factors prostaglandin F2 alpha (PGF2α) and prostaglandin E2 (PGE2), which are important for uterine contractions and cervical ripening (90).

On the other hand, some animal studies have shown the offspring of maternal Wistar rats and Sprague–Dawley rats fed a HFD have lower birth weights (91, 92). As a decreased arachidonic acid (ARA) concentration is related to delayed development of offspring (93), a lower birth weight may be related to impaired endogenous synthesis of arachidonic acid (ARA) in the DHA/EPA-supplemented group via impaired Δ6-desaturase expression (92).

Finally, some studies have shown that prenatal marine-based n-3 PUFA supplementation does not affect the birth weight of offspring (81, 94–96). No effects on birth weight were observed in Wistar rat offspring (94, 95). Similar to a study in the clinical setting, this intervention does not affect birth weight, length, or head circumference (96). Similar consequences were reported by Foster et al. (81).

### 5.3 Distant metabolic effects

#### 5.3.1 Alterations in lipid profile

DHA and EPA have TG-lowering effects. However, the cross-generational impacts of maternal DHA and EPA supplementation on lipid profiles in the offspring remain controversial in animal and clinical studies. In mice, a high-fructose diet (HFrD) induces dyslipidemia in offspring, characterized by hypertriglyceridemia and reduced plasma free fatty acid (FFA) levels. Maternal fish oil supplementation reverses HFrD-induced hypertriglyceridemia and low FFA levels, likely by increasing LPL activity to hydrolyze plasma TG. However, fish oil supplementation unexpectedly increases total cholesterol levels in offspring, potentially through the upregulation of the hepatic *de novo* lipogenesis genes 3-hydroxy-3-methylglutaryl-CoA reductase (Hmgcr) and 3-hydroxy-3-methylglutaryl-coenzyme A synthase 1 (Hmgcs1) (97). Similarly, another study revealed that plasma TG and unesterified fatty acid levels are reduced in Sprague–Dawley rats, supporting previous findings (92). In contrast, other studies have shown that maternal DHA enrichment during pregnancy and breastfeeding fails to attenuate elevated circulating TG levels in offspring fed a high-calorie diet (fcHFHSD) (16). A similar finding was observed in maternal HFD Wistar rats (95).

Human studies have shown inconsistent results. Maternal DHA supplementation may not consistently improve lipid profiles in the offspring. Compared with obese mothers receiving olive oil controls, obese mothers receiving fish oil capsules had offspring with lower TG levels but unaltered FFA levels at 3 months (80). However, another study revealed no impact of maternal DHA supplementation on nonfasting serum lipid, TG, cholesterol, HDL, or LDL in 4-year-old offspring (98). Additionally, no impact of prenatal n-3 LCPUFA supplementation on offspring TG or HDL levels at age 10 has been reported (99). Similarly, no differences in plasma LDL, HDL, total cholesterol, TGs, small dense LDL, or apolipoprotein levels were detected between 19-year-old offspring of mothers given n-3 LCPUFAs and those of mothers given olive oil (100).

#### 5.3.2 Obesity

The results of DHA and EPA supplementation on offspring obesity in animals and clinical trials are controversial (16, 83, 84, 94, 99). However, maternal supplementation with DHA and EPA during pregnancy and lactation appears ineffective in lowering the burden of childhood obesity in the population, although it may stimulate physical growth and development in offspring (80, 99). Studies remain inconclusive about the long-term effects on adiposity in offspring, with the overall evidence suggesting no beneficial impact on reducing obesity or overweight risk.

In animal studies, DHA and EPA do not affect body weight in 1-day-old or 21-day-old offspring (87) or in 3-month-old offspring (16). In clinical settings, the INFAT study revealed no variation in skinfold thickness, fat distribution, or abdominal fat mass in 1-year-old offspring (79) or in skinfold thickness, subcutaneous/visceral fat content, or fat percentage at 5 years of age between offspring of mothers receiving n-3 LCPUFA supplementation and control offspring (101). Similarly, a meta-analysis demonstrated no advantages of maternal n-3 LCPUFA supplementation on the risk of obesity in offspring, with BMI, BMI *z* scores, skinfold thickness, fat percentage, and fat mass being comparable to those of controls (84). Further studies reported that prenatal n-3 LCPUFA supplementation does not affect BMI or waist circumference in 19-year-old offspring (102), and no influence of DHA supplementation in mothers with high-risk pregnancies who are obese or have gestational diabetes was demonstrated on weight, height, BMI, arm circumference, or arm skinfold measurements in offspring at 2 or 4 years of age (81). Additionally, research has further confirmed that maternal consumption of DHA-rich fish oil during gestation has no effect on body composition or growth in 3 or 5 year-old children (103). Studies that assessed 5-year-old and 60-month-old infants presented similar results (78, 104).

By contrast, other studies have indicated that maternal DHA and EPA supplementation may promote physical development without increasing the risk of obesity. A meta-analysis revealed slightly elevated BMI z scores in 5- to 10-year-old offspring following maternal DHA and EPA supplementation during pregnancy (83). It was reported that fish oil supplementation in overweight/obese mothers led to increased ponderal indices and BMI z scores in 3-month-old infants, indicating stimulated growth without effects on fat percentage (80). In one cohort, weight, BMI, and skin-fold thickness were not affected in offspring (105). Additionally, 1- to 6-year-old offspring of mothers supplemented with n-3 LCPUFAs consistently presented a higher BMI, with increased total weight, bone mineral content, lean mass, and fat mass at 6 years, particularly lean mass, suggesting increased overall growth without elevated risk of obesity at this age (82).

However, gestational and lactational maternal DHA and EPA supplements increase the total body fat percentage, especially the subcutaneous fat mass, in rats (94). Clinical studies also show that longer-term follow-up studies have revealed potential adverse effects (99). It was suggested that metabolic health at 10 years demonstrated that offspring who are exposed to maternal n-3 LCPUFA supplementation have a greater average BMI, higher odds ratios for being overweight, elevated fat percentage, greater lean and fat mass, and a trend toward higher metabolic syndrome scores than controls, indicating possible detrimental effects (99). Although these findings may have been influenced by the 10-year dropout rate, despite the study’s high methodological quality, they warrant independent replication in future research (106).

#### 5.3.3 MAFLD

DHA and EPA intervention is efficacious for early-stage MAFLD, and the cross-generational effects of DHA and EPA supplementation on offspring in animal models, although not fully investigated in humans, are beneficial (16, 91). Prospective studies suggest that elevated maternal n-3 PUFA levels in pregnancy correlate with reduced hepatic fat content in offspring (107). However, this association between childhood hepatic fat accumulation appears to be significant only in males, potentially because of differences in n-3 PUFA metabolism or differential effects on gene expression between sexes (107).

Currently, the effect of maternal DHA and EPA exposure on the livers of offspring is less clear in humans, likely due to the limited supporting data, but most animal studies have demonstrated beneficial effects of maternal DHA and EPA supplementation. Offspring of DHA-supplemented obese rat dams exhibited lower hepatic TG levels, and this protective effect against MAFLD persisted until at least 6 months of age (16). It has been reported that maternal fish oil supplementation reduces liver TG levels in female offspring (91). Similarly, a decreased liver TG level was observed in male offspring following maternal fish oil supplementation (108). A reduction in liver TG levels in one-day-old pups has also been reported (92). It has also been reported that a superior impact of maternal supplementation results in a reduction in liver TG levels in 6-month-old offspring with free-choice, high-fat, high-sugar (fc-HFHS) diet-induced obesity and a partial amelioration of liver cholesterol levels in obese male offspring (16). With respect to liver injury, maternal fish oil supplementation has limited but sexually dimorphic effects on HFD-induced liver injury, with greater efficacy in females. At weaning, both male and female offspring of fish oil-supplemented mothers exhibit reduced mitochondrial damage in the liver. Although male offspring exhibit increased hepatic steatosis, ballooning, and inflammatory markers, female offspring exhibit no difference in steatosis compared with the HFD group; however, female offspring do exhibit reduced inflammation (95). However, some studies report no protective impact of maternal DHA supplementation on MAFLD in offspring, showing no alterations in the expression of most hepatic lipogenic genes (97).

## 6 Mechanisms of maternal DHA and EPA supplementation in transgenerational lipid metabolism

### 6.1 Transgenerational modifications in offspring adipose tissue caused by maternal DHA and EPA supplementation

**Figure 4:**
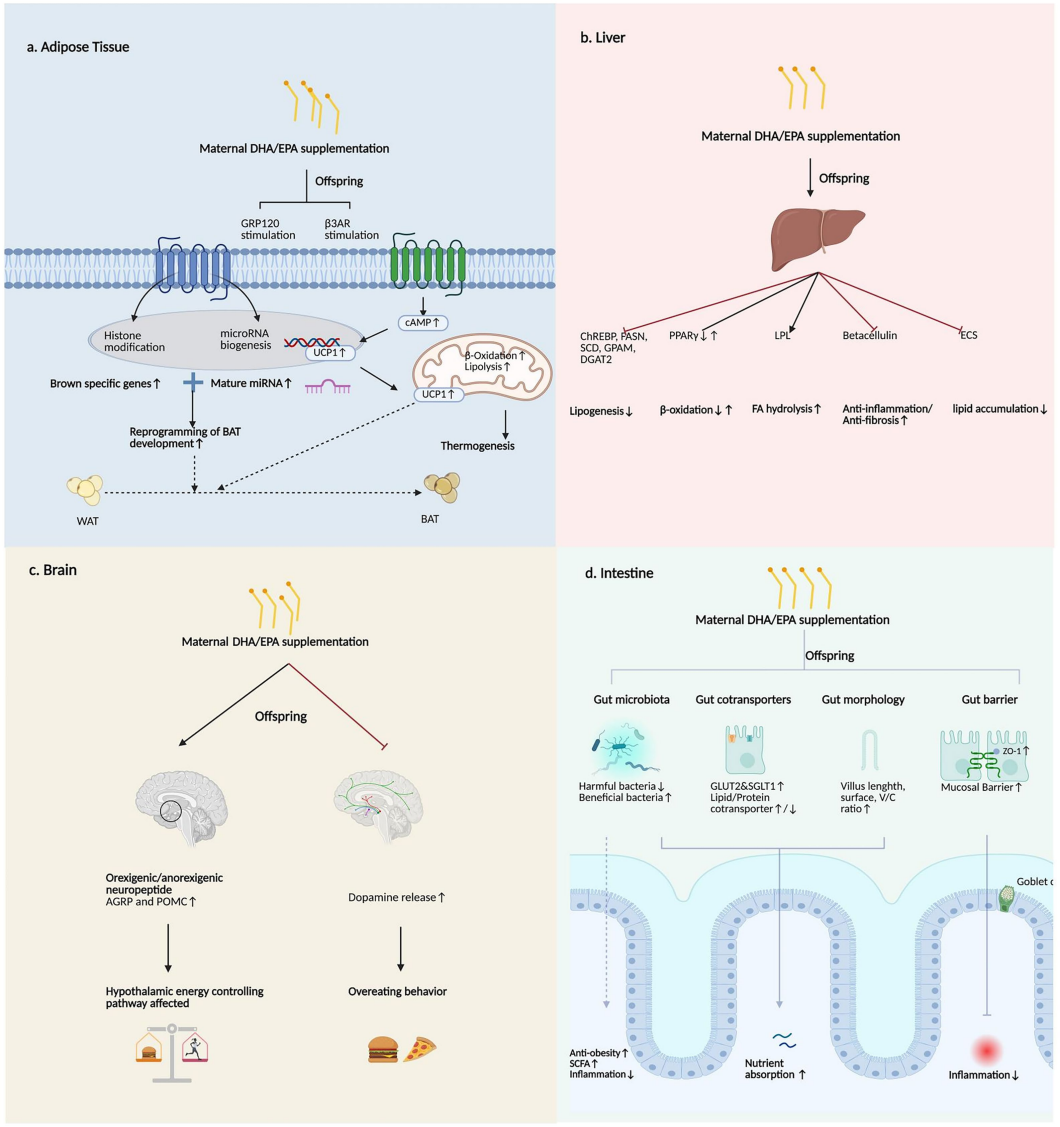
Mechanism of maternal DHA/EPA supplementation transgenerational effects on lipid metabolism, including alterations in adipose tissue, liver, brain, and intestine (figure created by https://www.biorender.com). **(a)** Adipose tissue. In brown adipose tissue, the membrane receptor GPR120 senses *n*-3 PUFAs, which enhances brown adipogenesis in offspring through epigenetic mechanisms involving histone modifications and miRNA biogenesis. Maternal DHA and EPA intake stimulates β3AR. β3AR stimulation is vital for β-oxidation and lipolysis. The activation of β3AR stimulates UCP1 expression through the cAMP signaling pathway. cAMP further regulates lipolysis and UCP-1-mediated thermogenesis. **(b)** Liver. Maternal DHA and EPA consumption inhibits offspring liver lipogenesis by regulating ChREBP, FASN, SCD, CPAM, and DGAT2. The effect of maternal DHA and EPA supplementation is still controversial in β-oxidation. Lipolysis-related LPL is upregulated in the livers of offspring following maternal fish oil intervention. Betacellulin overexpression is weakened in offspring exposed to high maternal DHA/EPA exposure, showing a therapeutic effect against inflammation and fibrosis in the offspring’s liver. Maternal fish oil supplementation has been observed to reduce ECS signaling, especially the protein content of CB1, inhibiting lipid accumulation in the offspring. **(c)** Brain. Feeding mothers a prenatal high-n-3 PUFA diet directly upregulates AGRP and POMC, affecting hypothalamic pathways controlling energy balance. Maternal DHA and EPA exposure inhibit dopamine release, thus inhibiting food consumption. **(d)** Gut: Maternal DHA and EPA supplementation benefits offspring gut microbiota composition by downregulating harmful bacteria and upregulating beneficial bacteria, upregulating SCFAs, and showing anti-obesity and anti-inflammation effects. This supplement also upregulates GLUT2 and SGLT1, which are two gut glucose cotransporters. Gut morphology also alters, showing increased villus length, surface, and V/C ratio, benefiting nutrient absorption. Gut barrier, including mucosal barrier and ZO1 enhanced, along with more goblet cells, showing anti-inflammation effects. DHA, docosahexaenoic acid; EPA eicosapentaenoic acid; GPR120, G protein-coupled receptor 120; miRNA, microRNA; β3AR, β-3 adrenergic receptor; UCP1, uncoupling protein 1; cAMP, cyclic adenosine monophosphate; ChREBP, carbohydrate response element binding protein; FASN, fatty acid synthase; GPAM, glycerol-3-phosphate acyltransferase; SCD, stearoyl-CoA desaturase; DGAT2, diacylglycerol O-acyltransferase 2; LPL, lipoprotein lipase; ECS, endocannabinoid system; CB1, cannabinoid receptor type 1; AGRP, agouti-related peptide; POMC, pro-opiomelanocortin; SCFA, short-chain fatty acid; GLUT2, glucose transporter 2; SGLT1, sodium/glucose cotransporter 1; V/C ratio, villus height/crypt depth ratio; ZO1, zonula occludens-1.

The mechanisms underlying the impacts of maternal DHA and EPA supplementation on transgenerational lipid metabolism involve multiple systems, like adipose tissue, the liver, the brain, and the gut (Figure 4). Research on maternal DHA and EPA supplementation-induced transgenerational changes in offspring WAT remains limited, and the fundamental mechanisms have been largely unexamined. For instance, studies indicate that a maternal EPA and DHA enriched diet reduces perirenal adipocyte size in offspring without affecting adipocyte number (87).

Unlike energy-storing WAT, BAT specializes in energy dissipation. In humans, the loss of active BAT depots contributes to the development of obesity (15). Importantly, EPA appears to be primarily responsible for the effects associated with adipocyte browning, whereas DHA has a negligible influence (109). Early stages of life are fundamental for fetal BAT development, with long-term consequences related to BAT function. Maternal n-3 PUFA intake stimulates fetal BAT development and upregulates uncoupling protein 1 (UCP1), PR domain-containing protein 16 (PRDM16), and GPR120 in the next generation (15). BAT induction of the expression of these brown-specific genes depends on the membrane receptor GPR120 that senses n-3 PUFAs. Fish oil intake upregulates both UCP1 and β3AR (110). Functionally, UCP1 serves as a key regulator during browning, while β-adrenergic receptor (β3AR) stimulation is vital for β-oxidation and lipolysis. DHA increases β-AR expression (111), which may actively affect its interaction with downstream adenylyl cyclase signaling, and this is vital for the recruitment of BAT (88). The activation of β3AR stimulates UCP1 expression through adenylyl cyclase signaling (112, 113). The plasma cAMP level is increased following maternal fish oil supplementation; cAMP is the downstream target of β3AR activation and regulates lipolysis and UCP-1-mediated thermogenesis (15). Thus, DHA and EPA may induce BAT recruitment and thermogenic responses through β3AR-UCP1 signaling. The offspring of maternal DHA- and EPA-supplemented mice exhibited upregulated expression of marker genes of beige and brown fat, both of which are related to energy expenditure (87). Such exposure also enhances thermogenic stimulators and potentially results in browning of WAT in offspring (87). Consequently, maternal n-3 PUFA supplementation may enable adipocytes within WAT to acquire BAT-like phenotypes, promoting energy dissipation through increased fatty acid oxidation in these depots (88).

Beyond direct gene regulation, maternal n-3 PUFA consumption enhances brown adipogenesis in offspring via epigenetic mechanisms that involve histone modifications and miRNA biogenesis (15). Placental transfer of n-3 PUFAs during pregnancy elevates its fetal levels, increasing cAMP concentrations and altering critical posttranslational modification (PTM) markers (H3K27Ac and H3K9me2) (15). This phenomenon accompanies epigenetic enzyme regulation, ultimately increasing the transcription of the BAT-specific genes PPARγ, UCP1, PRDM16, and peroxisome proliferator-activated receptor γ coactivator 1α (PGC1α) (15). Concurrently, histone acetylation increases transcriptome-wide miRNA gene transcription and upregulates Drosha, which is an RNA double-stranded ribonuclease, thereby promoting nuclear pri-miRNA processing and mature miRNA generation (114). Through this coordinated regulation via histone modifications and miRNA networks, n-3 PUFA strengthens fetal BAT transcriptional programming, potentially mediating long-term metabolic benefits later in life (15).

### 6.2 Transgenerational alterations in offspring livers caused by maternal DHA and EPA supplementation

Maternal supplementation with marine-based n-3 fatty acids alters hepatic lipid metabolism gene expression in the offspring. This protective effect of maternal DHA consumption on three-month-old offspring with MAFLD is linked to a decrease in the expression of hepatic mRNAs that encode proteins, including proteins that positively regulate lipogenesis and TG synthesis, the carbohydrate response element binding protein (ChREBP), fatty acid synthase (FASN), SCD, glycerol-3-phosphate acyltransferase, mitochondrial (GPAM), and diacylglycerol O-acyltransferase 2 (DGAT2) genes, as well as upregulation of genes supporting β-oxidation, such as PPAR-γ, CPT1a, peroxisome proliferator-activated receptor γ coactivator 1α (PGC-1α), medium-chain acyl-CoA dehydrogenase (MCAD), and hydroxyacyl-Coenzyme A dehydrogenase (HADH) (16). It is widely accepted that the protective capacity of DHA consumption on the MAFLD development is primarily achieved through the combination of inhibition of lipogenesis and the promotion of β-oxidation; for example, the expression of lipogenesis-related SREBP-1c is downregulated, that of β-oxidation-related CPT I, ACO, and HMG-CoA synthase is upregulated, and that of lipolysis-related lipoprotein lipase (LPL) is upregulated in the livers of offspring following maternal fish oil intervention (92). Similarly, the expression of the lipogenic markers Srebf1c and Fasn is decreased in rats following maternal fish oil-supplemented HFD feeding (91, 95). The expression of thermogenesis-regulated and β-oxidation genes is substantially elevated in the livers of offspring whose mothers were fed EPA- and DHA-enriched diets for 21 days versus the normal dietary group, facilitating hepatic lipolysis (87). However, maternal DHA supplementation leads to decreased expression of β-oxidation-related genes in the liver. This may occur because, on the one hand, fatty acid oxidation-related genes are already expressed in patients with MAFLD, with conflicting results, and on the other hand, whether increased lipid oxidation in MAFLD has protective effects is unclear. This result may also be related to the low level of EPA that was used in this study, as studies point out that EPA is more potent than DHA in increasing mitochondrial fatty acid oxidation (16).

Beyond lipid metabolism genes, maternal DHA/EPA supplementation modulates other hepatic pathways. The children of mothers on a high-sugar diet exhibit a marked upregulation in the expression of Δ-9 Desaturase, while its expression is fully restored to the control level by DHA and EPA supplementation, suggesting a protective effect (115). Similarly, Δ6-desaturase expression has been found to decrease in the liver (92). On a separate note, hepatic betacellulin, which is a growth factor, is overexpressed in those whose mothers have metabolic syndrome, and betacellulin overexpression is weakened in offspring exposed to high maternal DHA intake (115, 116). Studies have identified the suppression of betacellulin as a key mechanism through which DHA exerts its therapeutic effects against inflammation and fibrosis in metabolic dysfunction-associated steatohepatitis (MASH), and then, the effect of transgenerational changes in betacellulin levels may be a mechanism of action for inhibiting the development of MASH in the offspring (115).

Finally, the endocannabinoid system (ECS), which is associated with lipid accumulation and positive energy metabolism (117) via CB1 activation (91), is implicated in obesity and MAFLD (118). Maternal fish oil supplementation has been observed to reduce ECS signaling, especially the protein content of CB1 (91). These findings collectively advance the DOHaD paradigm by demonstrating that hepatic metabolic programming during critical windows of development may significantly influence lifelong susceptibility to metabolic disorders.

### 6.3 Transgenerational alterations in offspring appetite caused by maternal DHA and EPA supplementation

The impacts of maternal DHA and EPA supplementation on appetite of the offspring, especially the effects on the hypothalamic region, are controversial. Early in life, leptin promotes the development of hypothalamic satiety-regulating pathways, and high/low leptin levels early in life may increase the risk of future obesity (119). Agouti-related peptide (AgRP) is generated by specific neurons in the hypothalamic arcuate nucleus (ARC). AgRP further promotes appetite, reduces energy expenditure, and increases obesity by antagonizing the effects of alpha-melanocortin-stimulating hormone (α-MSH) (120). Pro-opiomelanocortin (POMC) is another precursor polypeptide that is expressed in the ARC, and its processing products include α-MSH, which suppresses appetite and promotes energy expenditure (121).

Leptin, AgRP, and POMC collectively influence the structure of the hypothalamic pathway of energy homeostasis and regulate the balance between appetite promotion and appetite suppression (122). Notably, feeding mothers a prenatal high-n-3 PUFA diet directly upregulates AGRP and POMC, which are important hypothalamic neuropeptides that regulate appetite and satisfaction in female offspring and do not depend on leptin (119). This change, however, does not occur in male offspring. Maternal n-3 PUFA-supplemented male offspring with low birth weight and low circulating leptin levels may exacerbate catch-up growth during the vital time window of growth and development, leading to a greater likelihood of subsequent metabolic disorders (119). Thus, n-3 PUFA supplementation may transgenerationally affect neuropeptide dysregulation and reduce leptin, which is deleterious to the metabolic and developmental outcomes of the next generation (119).

Additionally, low maternal consumption of n-3 PUFAs induces overeating behavior in the offspring (123). For example, offspring of pregnant mice having an n-6-high and n-3-low diet exhibit upregulated dopamine release, thus increasing food consumption (123). Overall, the impacts of supplemental maternal DHA/EPA intake upon offspring are varied and complex, and the existence of a benefit still needs to be supported by additional research.

### 6.4 Transgenerational alterations in the gut of offspring caused by maternal DHA and EPA supplementation

Maternal DHA and EPA may transgenerationally induce intestinal changes in the offspring, affecting gut microbiota structure, intestinal nutrient transport, morphological development, and barrier maintenance in early stages of life (17, 124). At birth, the gastrointestinal motility of newborns is immature, the activity of digestive enzymes is reduced, and the intestinal surface area for nutrient absorption is lower, preventing them from absorbing nutrients as effectively as adults (125). It is also important to distinguish between intestinal digestion (mechanical and enzymatic breakdown of foods), absorption (end products of digestion from the gastrointestinal tract into the blood and lymphatic vessels) and assimilation (effective physiological utilization), as the immature digestive tract of newborns may impose limitations on digestion and absorption, finally affecting the organism’s assimilation of nutrients (126, 127).

Maternal DHA and EPA supplementation promotes the upregulation of genes related to nutrient transport, thereby increasing nutrient transport capacity (17). N-3 LCPUFAs enhance nutrient absorption and assimilation, which aids in fulfilling the high nutritional demands for rapid growth and development in infants (125, 127). For instance, in weaning offspring from the maternal high-DHA group, genes related to carbohydrate transport sodium glucose co-transporter 1 (SGLT1) and glucose transporter 2 (GLUT2) were upregulated, potentially through the mTOR pathway, while lipid and protein transport genes were partly upregulated or downregulated (17). Nevertheless, proteomic and bioinformatic analyses of maternal high-DHA supplementation also demonstrated enhanced protein and fat absorption (17). In parallel, maternal DHA and EPA diets may improve the intestinal morphology of offspring, promote intestinal development, and may also protect the intestinal barrier by maintaining intestinal epithelial integrity and stimulating the expression of key genes and tissue morphology biomarkers to promote early intestinal health in offspring, thereby inhibiting the intergenerational passage of metabolic diseases (124). As the primary site of nutrient absorption, the morphology of the small intestine is crucial for providing adequate nutrition for growth and development (128). Villus height, surface, and the villus height-to-crypt depth (V/C) ratio are associated with nutrient transport, elevated in offspring subjected to a high-DHA maternal diet, indicating that supplementing the maternal diet with DHA during lactation improves intestinal nutrient transport in offspring under basal conditions (17). Though the actual assimilation and systemic utilization of these nutrients may increase as intestinal transportation increases, the overall efficiency of nutrient utilization remains lower than in mature individuals. This reflects the limitations imposed by the immature gastrointestinal tract in infants, despite an absolute increase in nutrient absorption.

In addition, research has indicated that maternal high-DHA intake leads to a downward trend in the *Firmicutes/Bacteroidetes* ratio in offspring, which is a microbiota marker associated with obesity (17); There are increased abundances of beneficial microbiota such as *Ruminococcus*, which is related to SCFAs production. *Lactobacillus* is associated with improving the intestinal barrier (17, 129). *Barnesiella* is associated with taurine-conjugated bile acids (17, 130). There are lower abundances of harmful or obesity-associated bacteria such as *Desulfovibrio*, *Alistipes*, *Acetatifactor*, *Oscillibacter*, *Harryflintia*, *Intestinimonas*, and *Pseudoflavonifractor* (17). These changes lead to significantly enhanced carbohydrate, fat, and protein digestion and absorption, as well as improved systemic metabolism, which benefits early growth and development (17). This demonstrates that maternal n-3 PUFA supplementation during pregnancy or breastfeeding may increase the abundance of metabolically beneficial microbiota while reducing the abundance of harmful or obesity-associated bacteria, and these changes in the microbiota may enhance systemic metabolism and promote early growth and development (17).

Finally, chronic low-grade inflammatory processes that drive obesity phenotypes allow for their perinatal transfer from mothers to their infants, which seems to be gut-initiated through the disruption of the intestinal barrier (131). Evidence indicates that maternal DHA and EPA intake may promote the expression of key genes and tissue morphology biomarkers to promote early intestinal health in offspring (124). A menhaden fish oil supplemented diet increases crypt length and goblet cell counts to increase mucus secretion (124). It also regulates ileal and proximal colon morphology, and upregulates the expression of antimicrobial proteins regenerating family member gamma (REG3γ), mediators like resistin-like molecule β (Relmβ), and zonula occludens (ZO-1), which play key roles in promoting mucosal barrier integrity, thereby helping to maintain mucosal integrity (124). Therefore, maternal DHA and EPA intake may protect the intestinal barrier in offspring during early life stages through intestinal immune regulation and inhibit the transgenerational transmission of metabolic inflammation (124, 131).

## 7 Individualized supplementation with EPA and DHA

### 7.1 Supplementation of DHA and EPA in offspring

After birth, offspring may obtain DHA and EPA through breastfeeding or formula feeding. To guide intake, the Food and Agriculture Organization (FAO) of the United Nations and the World Health Organization (WHO) proposed that the average prenatal and lactation nutrient requirement for DHA should be 200 mg/day and that the combined intake of DHA and EPA should be 300 mg/day (132). During gestation, when the RBC DHA level is below 5%, a daily intake of at least 600–800 mg DHA is advised (75). For 0–6 months-old infants, the recommended intake of DHA is 0.1–0.18% of overall energy intake, that is, about 40–130 mg/day (132).

In the case of formula feeding, it is also significant to focus on the levels of DHA and EPA in infant formula, and these levels should be similar to those in human milk. Current Chinese regulations stipulate that infant formula must contain at least 15 mg of DHA per 100 kcal, whereas the European Union regulations indicate a fundamental requirement of ≥20 mg of DHA per 100 kcal (133).

### 7.2 Maternal dietary patterns rich in DHA and EPA

Beyond direct supplementation, maternal diet is a modifiable and vital factor during the periconceptional period (134). Specific dietary patterns enriched with DHA and EPA during pregnancy represent promising nutritional interventions, with benefits observed for both maternal and metabolism in the offspring (135).

For instance, maternal adherence to a Mediterranean diet, characterized by moderate poultry and fish consumption, has been linked to reduced childhood abdominal obesity and improved neonatal metabolic profiles, including favorable lipoprotein levels, homocysteine concentrations, and insulin sensitivity (135). Similarly, the New Nordic Diet, which emphasizes fruits, vegetables, rapeseed oil, and fatty fish, is abundant in DHA and EPA (136). Likewise, the Okinawan diet in coastal populations ensures high n-3 LCPUFA intake (119), providing a natural model of n-3 PUFA enrichment (137). Notably, both the Okinawan diet and the New Nordic Diet have demonstrated efficacy in improving weight management and lipid homeostasis in patients with type 2 diabetes, underscoring their broader metabolic benefits (138).

Despite these promising findings, research on the transgenerational metabolic effects of these diets remains limited. Therefore, future research should examine how those dietary patterns affect lipid metabolism and metabolic health in offspring in the long run.

## 8 Conclusion and outlook

Fetal programming might influence the development of next generation, and in addition to genetic factors, intrauterine nutritional environments persistently influence fetal development. In particular, gestational and lactational marine-based n-3 fatty acid supplementation modulates lipid homeostasis in progeny, with potential long-lasting metabolic consequences. This review indicates that these nutritional interventions exert complicated and multidimensional effects on metabolism in the offspring, however, the mechanism remains incompletely understood because of the limited data available.

Several questions require more rigorous and detailed experimental designs to address. In clinical trials of DHA and EPA supplementation during pregnancy, the measured maternal omega-3 fatty acid levels may serve as an indicator to determine whether supplementation is adequate. Therefore, it is vital to assess maternal omega-3 status before supplementation (baseline), during the intervention period, and post supplementation to evaluate whether nutritional recommendations have been met. However, existing studies have not systematically monitored omega-3 levels across all three time points; most only measured levels at one or two stages. Consequently, relevant meta-analyses have generally not recorded or incorporated these longitudinal omega-3 status data from the included trials and have failed to perform subgroup analyses based on whether participants achieved sufficient omega-3 levels. This limits our ability to determine whether the observed effects of the supplement differ in efficacy among women who were initially deficient or sufficient. Also, the transgenerational impacts of DHA compared with EPA on offspring metabolic health is largely unexplored. In addition, long-term effects of maternal DHA/EPA exposure on offspring adulthood remain largely unknown in the clinical setting. Further studies are warranted to provide essential data to elucidate the transgenerational effects of DHA and EPA on metabolic health.

This review synthesizes recent evidence about the influence of maternal DHA and EPA supplementation on metabolic outcomes in offspring across critical developmental periods. During fetal development, maternal DHA and EPA supplementation appears to promote favorable metabolic programming by reducing white adipose tissue accumulation while increasing brown fat deposition and thermogenic capacity. With respect to birth outcomes, such supplementation has been associated with longer pregnancy time and heavier birth weight, a potentially beneficial effect that may reduce the chances of LBW and preterm delivery. However, while maternal DHA and EPA administration may be beneficial to the physical growth and development of offspring, it is not a compelling strategy for reducing childhood obesity. Current evidence about the impacts of maternal marine-based fatty acid supplementation on the lipid metabolism outcomes of progeny remains inconsistent. For example, studies reporting conflicting results on lipid profiles in the offspring preclude definitive conclusions. Interestingly, this contrasts with the well-established TG-lowering effects of DHA and EPA in adults, suggesting that their transgenerational impacts may not replicate the same plasma TG-reducing benefits.

Mechanistically, emerging research indicates that the transgenerational impacts of DHA and EPA could be mediated by promoting fetal adipose tissue browning, modifying hepatic lipid metabolism gene expression, impacting hypothalamic neuropeptide and feeding behavior, and altering the intestinal microbiota and barrier function. These findings jointly highlight the complex, multidimensional nature of the intergenerational metabolic programming induced by DHA and EPA.

More studies are needed to further assess maternal n-3 PUFA status and determine whether recommended intake levels are achieved, to investigate the distinct effects of DHA and EPA on offspring metabolic health, as well as to elucidate their roles in regulating metabolism of the next generation at different ages. If DHA and EPA may be applied as a clinical intervention to manage lipid metabolism in the offspring, it will be highly valuable for improving the well-being of offspring.
